# 

**Published:** 1889-12-21

**Authors:** 


### THE HOSPITAL

A WEEKLY JOURNAL OF
### Science, Medicine, Nursing & Philanthropy.

SATURDAY, DECEMBER 21st, 1889.

**The Registration of Nurses** - - - - - - 177
**Words of Consolation**—Goodwill towards Men - - 178
**The Doctor's Pulpit.**—A Suburban Medical Lecture - 179
**The British Nurses' Association and the Medical Council** - - - - - - - - - 180
**The Proposed Registration of Nurses** - - - 180
**M. Daudet's Darwinism** - - - - - - - 181
**Going Abroad** - - - - - - - - - 182
**Everybody's Page** - - - - - - - - 183
**Notes and News** - - - - - - - - 184
**Annotations.**—Mr. Speaker on "Humour and Fun"—Horses and Asphalte—Mr. Browning and Vivisection - - - 186
**Fallow Crops.**—Appeals - - - - - - - 191
**The Practitioner's Outlook and Retrospect:**
MEDICINE—Leprosy—Hysteria in the Male—Presystolic Murmur - - - - - - - - - 187
Renal Calculi—Alcoholic Paralysis - - - - 188
SURGERY—Surgical Dressings—Surgical Cases - - 188
LONDON EPIDEMIOLOGICAL SOCIETY—Malaria *v.* More Recognisable Causes of Disease - - - - - 189
NEW DRUGS, APPLIANCES, AND THINGS MEDICAL—The Derbyshire Respirator - - - - - - 189
**The Hospital Workshop.** No. XVIII.—King's College Hospital - - - - - - - - - 190
**The Student's Column.**—University of London - - 192
**Scraps and Gleanings** - - - - - - - 192

EXTRA SUPPLEMENT—THE NURSING MIRROR.

**En Passant.**—A Merry Christmas—Christmas Entertainments—Nurses for Wakefield—New Home at Middlesbrough—Lectures for Nurses—Short Items—Et Cetera, Et Cetera - - - - - - - - - xlix

**The Nurses' Bookshelf.**—Medicine in India—Minor Surgery and Bandaging - - - - - - - - l

**Notes on Opiates** - - - - - - - - l

**Notes from Australia** - - - - - - - li

**Everybody's Opinion.**—Certificates and Registration - li

**Notices—Death in Our Ranks** - - - - - lii

**Appointments—Notes and Queries** - - - - lii

**Vacancies—Amusements and Relaxation** - - - lii

